# Expression and Regulation of the *Escherichia coli* O157:H7 Effector Proteins NleH1 and NleH2

**DOI:** 10.1371/journal.pone.0033408

**Published:** 2012-03-12

**Authors:** Ashleigh Holmes, Cecilia S. Lindestam Arlehamn, Dai Wang, Tim J. Mitchell, Tom J. Evans, Andrew J. Roe

**Affiliations:** Institute of Infection, Immunity and Inflammation, College of Medical, Veterinary and Life Sciences, University of Glasgow, Glasgow, United Kingdom; Montana State University, United States of America

## Abstract

**Background:**

*E. coli* O157 carries two genes encoding the effector proteins NleH1 and NleH2 which are 87% identical. Despite the similarity between the proteins, the promoter regions upstream of the genes encoding the effectors are more divergent suggesting that the actual expression of the genes may be differentially regulated. This was tested by creating reporter fusions and examining their expression in different genetic backgrounds, media and on contact with host cells. The function of the proteins was also tested following transfection into host cells.

**Principal Findings:**

Expression of both NleH1 and NleH2 was enhanced when cultured under conditions that stimulated expression of the Type Three Secretion System (T3SS) and was influenced by the regulators Ler and GrlA. Maximal expression of NleH1 required 531 bp of the upstream untranslated region but NleH2 required only 113 bp. Interestingly, contact with host cells strongly repressed expression of both NleH1 and NleH2. Following transfection, both proteins produced only minor effects on NF-κB activation when assessed using a NF-κB luciferase reporter assay, a result that is consistent with the recent report demonstrating the dependence on RPS3 for NleH1 modulation of NF-κB.

**Significance:**

This study demonstrates the importance of considering gene regulation when studying bacterial effector proteins. Despite their sequence similarity, NleH1 and NleH2 are expressed differentially and may, therefore, be translocated at distinct times during an infection.

## Introduction

Enteropathogenic *Escherichia coli* (EPEC) and enterohaemorrhagic *E. coli* (EHEC) are important causative agents of infectious diarrhoea worldwide. EPEC is the leading cause of prolonged watery diarrhoea in children living in developing countries [1]. EHEC causes sporadic outbreaks of haemorrhagic colitis and haemolytic uremic syndrome that have been widely reported in Europe, North America and Japan. EPEC and EHEC are extracellular pathogens that mediate an initial attachment via adhesins, such as their flagella [2], [3], prior to a more intimate attachment. This intimate attachment is characterized as an attaching-and-effacing (A/E) lesion due to destruction of the brush-border microvilli and cytoskeletal rearrangements to form pedestals; this is dependent upon the expression of a Type Three Secretion System (T3SS).

The genes which encode the T3SS machinery, translocators, effectors, chaperones and its own regulators are within the Locus of Enterocyte Effacement (LEE) pathogenicity island (PAI). The T3SS apparatus not only translocates the 7 LEE-encoded effectors such as Tir [4], Map, and EspF but also other proteins encoded on prophage elements throughout the genome, which are termed non-LEE encoded effectors (Nle) [5], [6], [7], [8], [9].

Horizontally acquired genetic elements require appropriate regulation of expression that can be managed by both endogenous and exogenous elements. Currently, it is understood that expression of the LEE PAI is tightly regulated by an interplay of LEE-encoded, global and other horizontally-acquired regulators (reviewed in [10], [11]). The LEE encodes three regulatory elements; LEE-encoded regulator (Ler), Global Regulator of the LEE Activator (GlrA) and Grl Repressor (GlrR). *Ler* is the first open reading frame of *LEE1* and belongs to the H-NS family of nucleoid-associated proteins which positively regulates transcription of both LEE and non-LEE genes [12], [13]. Ler activates gene transcription by counteracting the effects of global regulator H-NS which silences transcription of genes by binding to curved AT-rich regions [14]. H-NS has been shown to repress the transcription of *ler* and *LEE4* [15], [16]. GrlA and GrlR are encoded between LEE1 and LEE2, are co-transcribed and transcription of these genes is dependent upon Ler [16], [17]. GrlA can in turn positively regulate the expression of Ler through interacting with the LEE1 promoter [18] forming a positive feedback loop [19]. GrlR directly interacts with GrlA and this interaction is proposed to act as a check-point to downregulate the feedback loop [18], [19], [20]. Additional horizontally acquired elements that regulate the LEE include PerC (plasmid encoded regulator C) in EPEC or PerC-like homologues (Pch) in EHEC, of which there are seven present in the genome. PchABC can act globally by enhancing the transcription of *LEE1* (*ler*) and non-LEE encoded genes both dependent and independent of Ler [21], [22], [23].

Non-LEE encoded effector H (NleH) was identified as a homologue of the *Shigella flexneri* effector OspG [7], a serine-threonine protein kinase which subverts the host innate immune response [24]. NleH is conserved amongst the A/E pathogen family with *Citrobacter rodentium* encoding one allele and EPEC and EHEC encoding two alleles [7], [25], [26]. Despite the protein similarities of *E. coli* O157 NleH1 and NleH2 being greater than 80%, we found that their putative promoter sequences were more divergent and less well conserved, suggesting possible differences in their regulation and expression. The regulation of expression of *C. rodentium* NleH (CRODNleH) has been demonstrated to be largely post-translational, dependent upon the Ler/GrlA regulon [25]. However, as the upstream sequence of CRODNleH is only ∼50% similar (see Table S1) to that of EHEC NleH1 (z0989) and NleH2 (z6021) we wished to investigate the control of NleH1 and NleH2 gene expression. We also investigated if EHEC NleH1 and NleH2 were under the regulatory control of Ler and/or GrlA.

## Results

### *In vitro* expression of *nleH1*::*gfp* and *nleH2*::*gfp* in *E. coli* O157:H7

Our previous work performed transcriptional profiling of the global changes associated with induction of the LEE, the genes that encode the *E. coli* O157:H7 T3SS. We have previously reported that MEM-HEPES media provides excellent conditions for expression of the LEE, even when compared to the widely used DMEM media. For example *escJ* and *escN*, which encode basal apparatus proteins, were both strongly induced in MEM-HEPES data. Analysis of the same transcriptomic data showed that *nleH1* showed a 6.4 fold (p = 0.008) change in expression when cultured in MEM-HEPES when compared with DMEM (accession no. GSE6296). Therefore, *nleH1* was clearly expressed *in vitro* and subject to transcriptional regulation. We aimed to determine if *nleH1* and *nleH2* were expressed at the same time during growth in liquid media and following contact of *E. coli* O157 with host cells.

**Figure 1:**
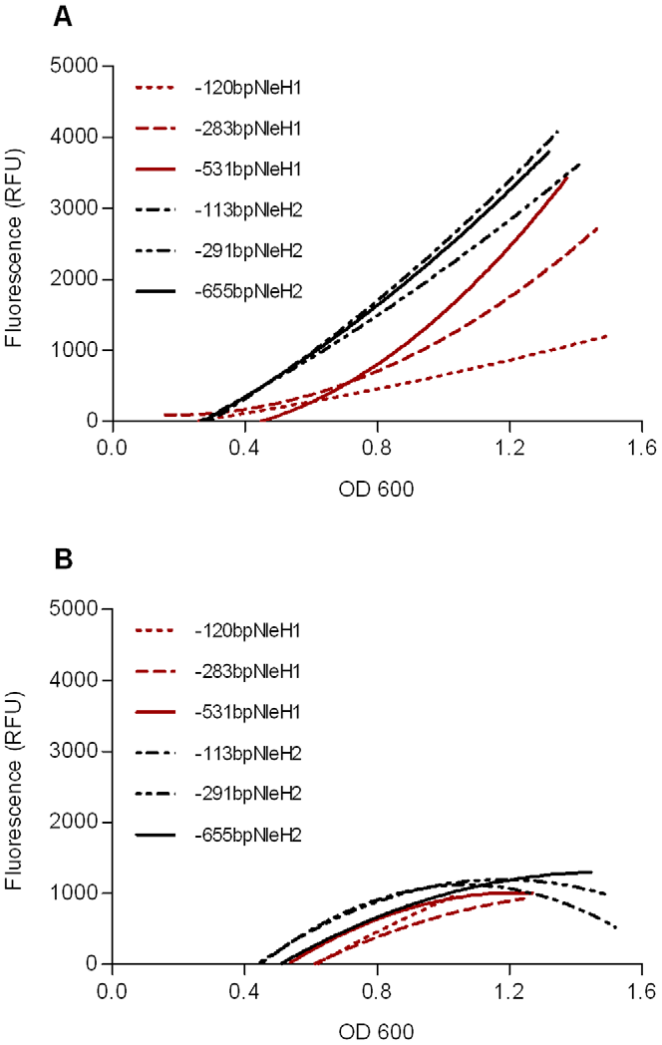
Expression of NleH-GFP constructs in *E. coli* O157:H7 grown in defined media. Constructs consisting of 120 bp (pAHE18), 283 bp (pAHE19) or 531 bp (pAHE8) of the NleH1 5′ UTR and 113 bp (pAHE20), 291 bp (pAHE21) or 655 bp (pAHE22) of the NleH2 5′ UTR cloned upstream of *gfp* were transformed into ZAP193, grown in MEM-HEPES (A) or DMEM (B) and GFP fluorescence measured during growth. All values were corrected for background from a promoter-less GFP (pAJR70) control measured at the same optical density. Graphs represent the average of three experimental repeats.

Upon assessment of the untranslated region (UTR) upstream of *nleH1* and *nleH2*, we noted that the first 100 bp were 70% identical, with this figure falling to 50% identity when extending this region to 500 bp upstream of the ATG start codon (Figure S1). To examine if these differences affected *nleH1* and *nleH2* expression and regulation, we generated a series of promoter fusions consisting of different lengths of the upstream UTR of the two genes fused to *gfp*. In each case, the complete *nleH1* or *nleH2* coding sequence was included in the fusion to create a “full-length” translational fusion. Each plasmid was transformed into *E. coli* O157:H7 and the amount of GFP produced monitored during growth in MEM-HEPES (Figure 1A) or DMEM media (Figure 1B). Highest expression of *nleH1*::*gfp* was achieved with the fusion containing the longest upstream UTR regions: 531 bp (pAHE8) (Figure 1). Expression of NleH2 was less dependent on promoter length, requiring only 113 bp (Figure 1A). When cultured in MEM-HEPES media, the *nleH1*::*gfp* and *nleH2*::*gfp* fusions showed markedly higher expression compared when cultured in DMEM: at an OD_600_ = 1.2, both fusions gave four-fold higher expression in MEM-HEPES compared to DMEM (Figure 1A and B). As the UTR was reduced in MEM-HEPES, *nleH1*::*gfp* showed a step-wise decrease in the amount of expression, with the fusion driven by the 120 bp UTR (pAHE18) producing less than 25% of the GFP compared to the 531 bp UTR fusion (pAHE8; Figure 1A). In comparison, expression of *nleH2*::*gfp* was largely unaffected: reducing the length of the UTR to 113 bp (pAHE20) still produced 90% of the expression compared to the fusion containing the 655 bp UTR (Figure 1A). These results suggest that expression of *nleH1* is subject to stricter control through the influence of transcription factors and/or secondary DNA structure compared to *nleH2*, and that this control depends upon 120–531 bp of the 5′ UTR.

### Expression in different genetic backgrounds

**Figure 2:**
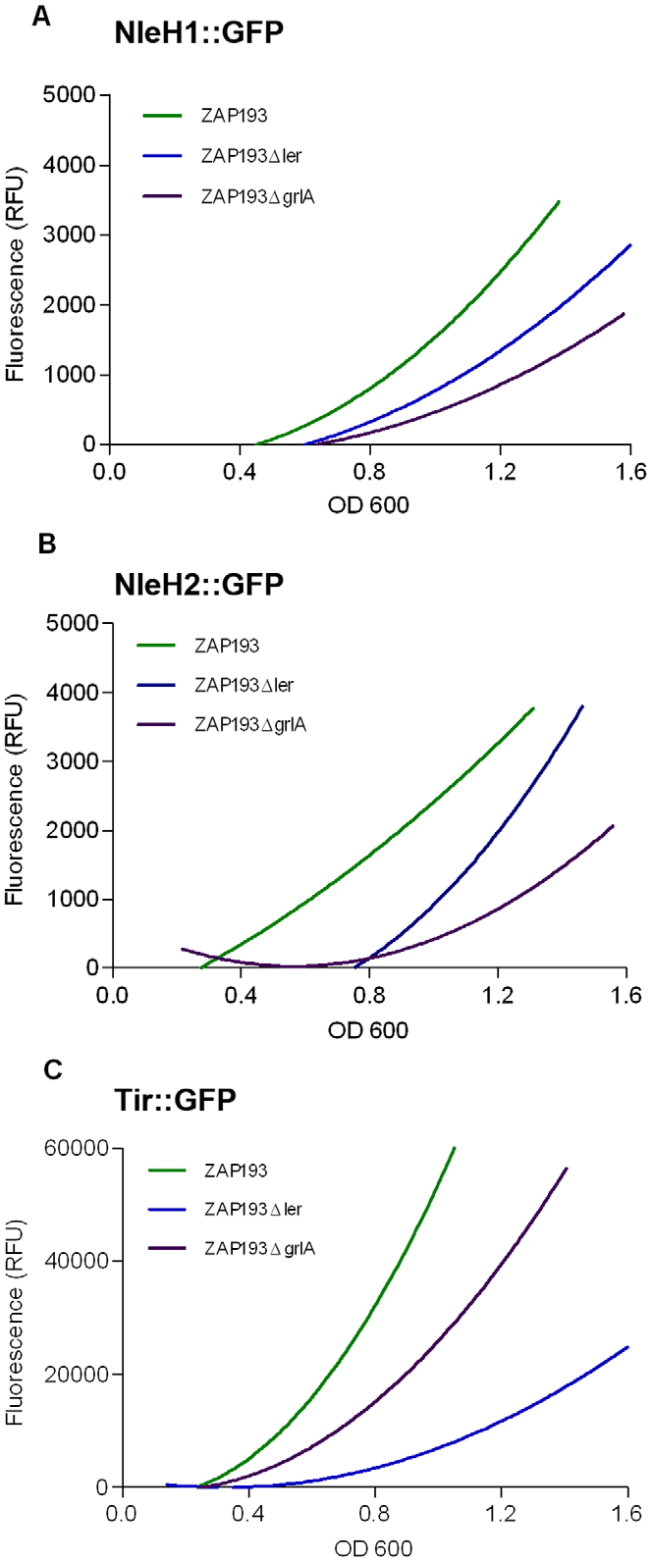
Expression of NleH-GFP and Tir-GFP in *E. coli* O157:H7 defined LEE regulator mutants. *E. coli* O157:H7 ZAP193, ZAP193Δ*ler* and ZAP193Δ*grlA* were transformed with constructs expressing NleH1-GFP (pAHE8; A), NleH2-GFP (pAHE22; B) and Tir-GFP (pAJR132; C). GFP expression was monitored during growth of the transformants in MEM media, with a promoterless GFP construct (pAJR70) as a background control. Fluorescence values were corrected for background and lines represent the average of three biological repeats.

The data in Figure 1 showed maximal expression of *nleH1* and *nleH2* at high optical densities raising the possibility that either quorum sensing mechanisms or stationary phase sigma factors may contribute to their regulation. To test this, we transformed the *nleH1*::*gfp* and *nleH2*::*gfp* reporter plasmids, pAHE8 and pAHE22 into *E. coli* K12 and isogenic deletion strains of *rpoS* and *qseC*. Expression was tested in a number of K12 strains, including BW25113 and MC4100, but in all cases expression was negligible implying that an O157-specific regulator may be essential for their production (data not shown). We then assessed the importance of the LEE-associated regulators, Ler and GrlA for *nleH1* and *nleH2* expression. Plasmids pAHE8 and pAHE22 were transformed into *E. coli* O157:H7 strain ZAP193, an isogenic *ler* deletion strain and ZAP193 containing a mini-Tn5 cassette insertion in the *grlA* gene. Expression of *nleH1* and *nleH2* was markedly reduced by deletion of either *ler* and *grlA* (Figure 2A–B). At an OD_600_ = 1.0, *nleH1* expression fell by 50% and 70% when *ler* or *grlA* were deleted respectively (Figure 2A). For *nleH2*, the reduction was 50% and 80% in the same backgrounds (Figure 2B). As a control, we also used a *tir*::*gfp* fusion, pAJR75 (Figure 2C) consisting of the *LEE5* promoter region. This fusion also showed *ler* and *grlA* dependence for full expression of *gfp* but was expressed at much greater levels than *nleH1* and *nleH2*, typically 15-fold higher.

**Figure 3:**
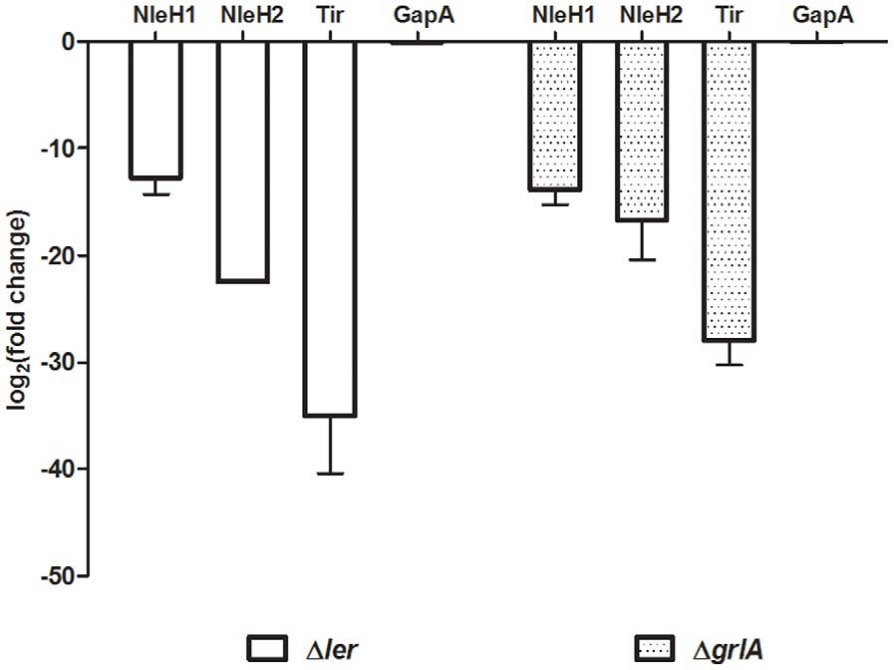
Quantitative PCR of NleH transcripts in LEE regulator knockouts. RNA was collected from ZAP193 strains WT, Δler and ΔgrlA grown to OD_600_ = 1.2 in MEM and cDNA prepared. NleH1, NleH2, GapA, Tir and 16S RNA transcript was then quantified by q-PCR, NleH values normalised to that of 16S RNA, and the fold change calculated comparing mutant to wild-type. Bars represent the average of three biological samples. Error bars represent the standard error of the mean.

To confirm the regulatory importance of *ler* and *grlA* on *nleH1* and *nleH2* expression, Q-PCR was used to assess levels of transcription directly. Strains were cultured in the same MEM-HEPES media as for the previous assays and cDNA prepared as described in Materials and Methods. Transcription of both *nleH1* and *nleH2* was markedly affected in the Δ*ler* background, falling greater than ten-fold (Figure 3). Similarly, deletion of *grlA* reduced transcription of both *nleH1* and *nleH2*. The housekeeping gene, *gapA*, encoding glyceraldehyde-3-phosphate dehydrogenase showed only very minor changes in the different regulatory backgrounds (Figure 3). Expression of the gene encoding Tir, was found to be highly dependent on both Ler and GrlA confirming that both regulators are critical for expression of LEE-encoded effector proteins. Overall, these results confirm that *nleH* expression and transcription is dependent upon the presence of Ler and GrlA, as seen previously with the GFP reporter fusions (Figure 2A–B).

### Single cell expression

**Figure 4:**
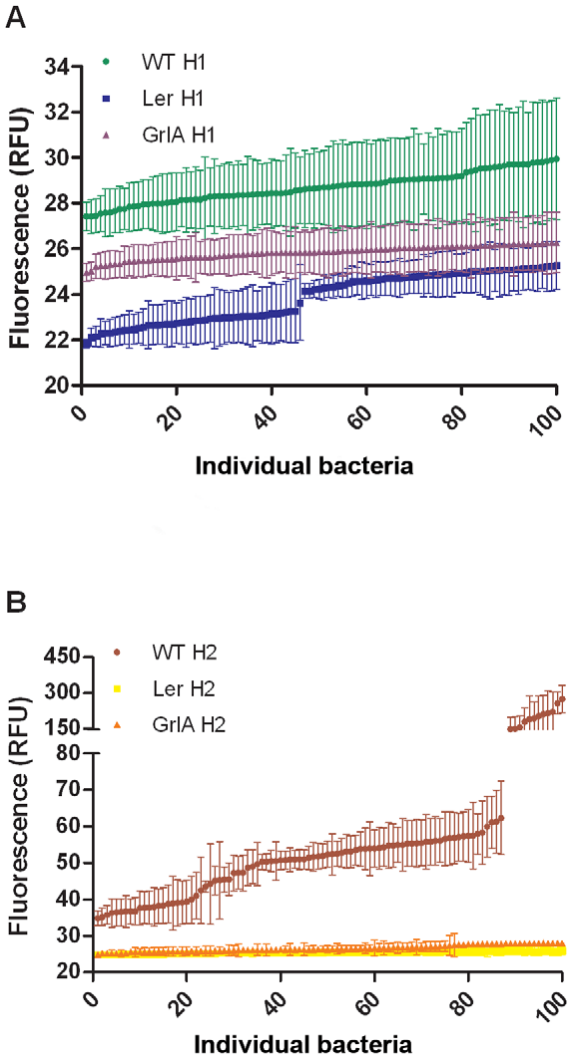
Fluorescence microscopy of NleH-GFP. pAHE8 (NleH1-GFP) and pAHE22 (NleH2-GFP) were transformed in ZAP193, ZAP193Δler and ZAP193ΔgrlA and at OD_600_ = 0.8, dried onto a microscope slide in 4% PFA and stained for EspA filaments. Volocity quantification software was used to determine the average GFP fluorescence per voxel of 100 individual bacteria for NleH1 (A) and NleH2 (B). Each point represents the average GFP fluorescence from a composite from 16 z-slice images thus reducing planar effects. Error bars represent the standard deviation.

Previous work has demonstrated that several LEE-encoded and Nle effectors can be heterogeneously expressed within a population when assessed by either reporter fusions or by indirect immunofluorescent imaging. To determine if NleH1 and NleH2 expression was homo- or heterogeneous, bacteria transformed with pAHE8 and pAHE22 were examined using fluorescence microscopy and the expression of a minimum of 100 bacteria from at least three fields quantified using Volocity suite software (Perkin-Elmer). Expression of NleH1 was uniform, with the population expressing an average of 29 (±1) RFU (Figure 4A). In comparison, it was clear that NleH2 expression was more heterogeneous, with the majority of bacteria (86%) expressing an average of 49±8 relative fluorescence units (RFU) but a small population (14%) expressing an average of 234±55 RFU (Figure 4B). When expression was assessed in Δ*ler* or Δ*grlA* backgrounds, expression was lower for both NleH1 and NleH2 and the heterogeneity for NleH2 was no longer detectable. To ascertain whether NleH was expressed in co-ordination with the T3SS apparatus, the EspA filaments were immunostained as described previously [13]. The results show NleH expression is not strictly co-ordinated with the LEE as 18–20% of the population does not express EspA, but still express NleH (Figure S2A). Also, this percentage of EspA negative cells is maintained within the heterogenous population of NleH2-GFP expressing cells. As expected, EspA filaments are not detected in a *ler* negative background and are reduced in the *grlA* mutant (Figure S2B–C).

### Expression of *nleH1*::*gfp* and *nleH2*::*gfp* in *E. coli* O157:H7 upon contact with host cells

**Figure 5:**
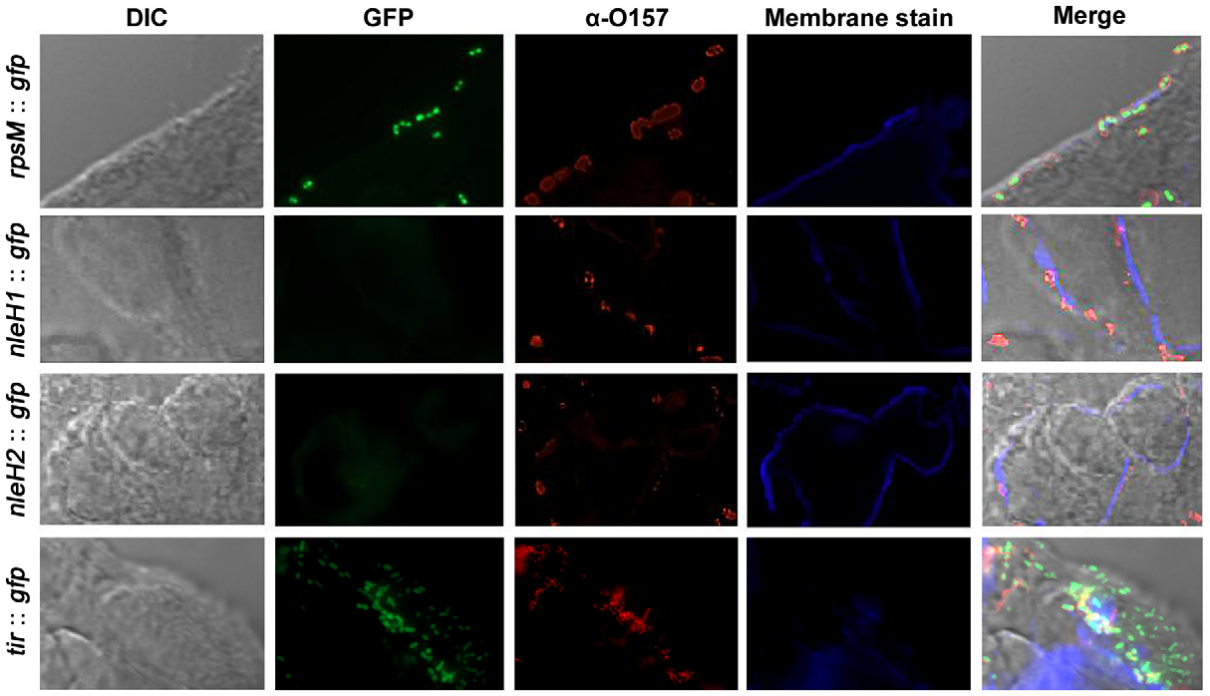
Expression of NleH-GFP upon *E. coli* O157:H7 ZAP193 contact with EBL cells. ZAP193 transformed with plasmids expressing GFP constitutively (pAJR145; *rpsm*::*gfp*) or translational fusions of *nleH* or *tir* to *gfp* under the control of their native promoter (pAHE8; NleH1-GFP, pAHE22; NleH2-GFP, pAJR75; Tir-GFP) were added to EBL cells and incubated for 0, 5, 30, 60 or 180 minutes at 37°C, 5% CO_2_ before the removal of supernatant and fixation of cells with 4% paraformaldehyde. The panel of images is representative of all time points tested, apart from Tir-GFP, that showed strong early expression during cell contact but was markedly reduced at 180 minutes.

The expression of effector proteins is subject to strict regulation thereby producing a discrete and carefully orchestrated pattern of protein injection into host cells. To test the regulation of *nleH1* and *nleH2* upon contact with host cells, we examined the expression of pAHE8 and pAHE22 during the interaction of *E. coli* O157:H7 with bovine epithelial cells (EBL). Bacteria were initially cultured to an OD_600_ = 0.6 in MEM-HEPES media to promote expression of the T3SS before addition to the cell line at a multiplicity of infection of 200. When cultured in this media, we had previously seen rapid onset of GFP expression from these plasmids as optical density increased. Bacteria in contact with host cells were detected using an anti-O157 specific antibody and appropriate secondary conjugate. We examined expression at time points 5, 30, 60, 180 and 420 minutes after initial contact but no expression from *nleH1* or *nleH2* could be detected, suggesting marked repression of expression (Figure 5). In contrast, the control plasmid consisting of the promoter from the gene encoding the small ribosomal subunit (*rpsM*) fused to *gfp* (pAJR145) gave consistent and readily detectible expression throughout the course of the experiment. We also used a previously characterized *tir*::*gfp* fusion (pAJR75) and this gave rapid early expression during contact with the EBL cell line. Tir expression and was evident 60 mins after initial cell contact (Figure 5) but expression was reduced after that timepoint (data not shown).

### Effect of NleH1 and NleH2 on host cell inflammatory response

**Figure 6:**
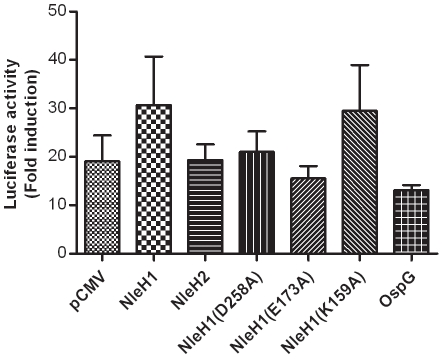
NF-κB activity in the presence of NleH variants. HEK293T cells were co-transfected with a luciferase reporter plasmid under the control of consensus κB sites, a β-galactosidase plasmid and a control (pCMV), NleH or OspG vector. After 40 hours, cells were stimulated by the addition of TNF-α (25 ng/ml; 24 hours). Results represent three biological replicates, where variants were tested in triplicate and assayed in duplicate. Statistical analysis with one-way ANOVA shows no significant difference compared with the pCMV control. Error bars represent the standard error of the mean.

The effect of NleH1 and NleH2 on host cell NF-κB modulation was then tested. Both proteins share a conserved domain with serine/threonine protein kinases including the Shigella effector protein OspG, which has been reported to strongly inhibit the activation of NF-κB [24]. To test the role of this kinase activity, site directed mutants in three key residues were created. Specifically, lysine 159, aspartate 258, and glutamate 173 were substituted for alanine residues using site-directed mutagenesis. A K159A substitution results in loss of NleH1 kinase activity [27], [28] and was confirmed by *in vitro* kinase assays with recombinant NleH1 protein (results not shown). Mutants D258A and E173A were created as these are highly conserved residues in Ser/Thr protein kinases and therefore may play important roles in their function. Each vector was transfected into a HEK293T cell line [29] alongside an NF-κB luciferase reporter plasmid and a constitutively expressed β-gal control plasmid. The β-gal activity was used to control for any variations in transfection efficiency and to normalize between replicates. Cells were stimulated using 25 ng.ml^−1^ TNF-α for 24 hours and the fold increase in NF-κB activity was determined using a luciferase reporter assay. The vector control gave a 20-fold stimulation of NF-κB activity upon addition of TNF-α demonstrating that the cells were responding as expected. Transfection with the vector expressing NleH1, NleH2, OspG or any of the site-directed variants produced no significant differences in the level of NF-κB activation as tested by one-way ANOVA (Figure 6). Although not statistically significant in our assay, OspG does show a trend towards repression of NF-κB activation, which was previously reported [24].

## Discussion

Over the past ten years, the repertoire of *E. coli* O157 T3SS effector proteins has expanded greatly. This can be attributed to improvements in the sensitivity of mass spectrometry instruments and some excellent bioinformatics based studies, revealing a potential suite of over 50 effector proteins. Many of the identified Nle effector proteins can be grouped due to their high levels of identity, for example the EspG “family”. However, understanding the temporal expression and function for each of these effectors, in addition to how they might function co-operatively or antagonistically, presents a daunting challenge for researchers.

In the current study, we examine the expression and regulation of *nleH1* and *nleH2* from *E. coli* O157:H7, which show a high level of similarity but greater diversity in their upstream UTRs, providing the potential for differential gene expression. Our previous work demonstrated that *nleA*, *nleB*, *nleD*, and *nleE* are transcribed in *E. coli* O157:H7 under secretion-permissive conditions [13]. Using GFP fusions, Q-PCR analysis and through interrogation of existing microarray data, we found that both *nleH1* and *nleH2* were expressed when grown in tissue culture medium. Maximal expression of *gfp* reporter fusion to *nleH1* required the longest upstream UTR region consisting of 531 bp. *nleH1* expression was more dependent on UTR length compared to *nleH2*. One interpretation of these data is that a transcriptional activator may bind strongly in a region further upstream of the −283 bp 5′ UTR of *nleH1*, and potentially at a similar site for *nleH2*, albeit much more weakly. Using ChIP-on-chip analyses, recent work demonstrated that the EHEC O157 Per-C like homologue (Pch) regulator directly binds the *nleH1* promoter but binds further upstream in the *nleH2* prophage in order to exert its control [21]. This study also determined that Pch does not have a consensus binding site, as it has various numbers and positions of binding sites depending upon the target gene, thus regulating a broad range of genes. Therefore, the increase in NleH1-GFP expression with increase in 5′ UTR may be due to a direct effect of Pch binding to the area between −283 and −531 bp or an indirect effect of Pch modulating the promoter's secondary structure. This in turn may facilitate the action of another positive regulator or displace a repressor such as H-NS.

Assessment of the translational fusions in LEE-encoded regulator, Ler and GrlA deletion strains of *E. coli* O157:H7 ZAP193 indicated that expression of NleH1 and NleH2 requires these regulators. Expression was reduced greater in the absence of GrlA compared to Ler. These data are interesting to compare with a previous study investigating NleH regulation in *C. rodentium* [25]. This study also reported reduced expression of the NleH fusion in *C. rodentium* Δ*grlA* and Δ*ler* mutants compared to wild-type, albeit not significantly in this organism. In the current study, both *nleH1* and *nleH2* were subject to transcriptional regulation as demonstrated by Q-PCR analyses that showed that *nleH1* and *nleH2* transcripts were reduced approximately four -fold in the absence of *ler* or *grlA*. Therefore Ler and GlrA both influence *nleH1* and *nleH2* expression in both *E. coli* O157 and *C. rodentium*, but to markedly different extents. Further work needs to be performed to elucidate whether these LEE encoded regulators directly interact with the UTR of *nleH1* and *nleH2* or indirectly influence expression via other transcription factors.

Many *E. coli* O157:H7 virulence factors are expressed heterogeneously, such as EspA [30], Tir, Map, intimin [31] and NleA [13], in order to co-ordinate expression of the effectors with that of the T3S apparatus. Single cell imaging of NleH-GFP expressing *E. coli* O157:H7 showed that NleH-GFP is expressed by all cells in the population. However, the fluorescence of GFP measured on a per cell basis is homogenous in NleH1 but heterogeneous in NleH2. When cultured in MEM, only 80% of the population co-stained for EspA filaments, correlating with previous reports showing that only a subpopulation of ZAP193 (40–80%) express EspA filaments when cultured in the same media [32]. This percentage was maintained in the ‘hyperexpressor’ population of NleH2. This shows that although NleH-GFP expression is induced by the same conditions as that for the LEE, it is not strictly co-ordinated. In comparison, expression of *nleA* has been shown to be closely co-ordinated with the LEE and its transcription has been shown to be directly regulated by Ler [13], [21], [33]. NleA also plays an important role in the virulence of A/E pathogens [34], [35].

The data show that both *nleH1* and *nleH2* are likely to be repressed during the early stages of A/E lesion formation and may be required later in the infection process. NleH-GFP expression was not detected upon contact with host cells under the conditions tested, and it has previously been reported that host cell contact results in a reduction in expression of many non-LEE encoded genes [13], [36]. The persistence of *E. coli* O157:H7 on host cells has been shown to be mediated through the transcriptional regulator GadE [11], [37]. The induction of the GAD stress response and reciprocal repression of the LEE mediated by GadE is, in turn, controlled by the *psr* genes. It has been reported that there is a high association between non-LEE encoded effectors with *psr* and/or *pch* regulated genes encoded on the same horizontally acquired element [11]. This association led to the hypothesis that the Psr mediated induction of *gadE* transcription and subsequent repression of LEE encoded effectors facilitates non-LEE encoded effector secretion [11]. It is interesting to note that many Nle effector proteins, including NleH, exhibit a role in colonisation rather than A/E lesion production or overt pathogenesis [25], [26], [38], [39], [40], [41], [42], [43].

NleH is a predicted Ser/Thr protein kinase, and the C-terminal end of the protein shares sequence similarity with that of the *S. flexneri* effector OspG. OspG has been reported to modulate host innate immune responses by interfering with NF-κB activation [24] with a 70% reduction in NF-κB activation being observed when five times more OspG (0.5 µg) than the NF-κB reporter (0.1 µg) was transfected into HEK293T cells. This study reported a more modest 30% decrease in NF-κB activation when equivalent concentrations of OspG and reporter plasmid were used. This is broadly comparable to the level of NF-κB inhibition we observed (∼17%) using OspG when equivalent concentrations of effector and NF-κB reporter plasmids were transfected (0.4 µg).

During the course of this study, it was reported that NleH1 inhibits NF-κB activation [27], [44]. Luciferase reporters were transfected into HEK293T cells in a manner similar to that which was carried out in this study. However,cells were stimulated with four times more TNF-α and analysed 1 hour post-stimulation. Similar to the OspG study [24], the effector plasmid and the NF-κB reporter plasmid were transfected at a 4∶1 ratio which appears to amplify the effect (to a similar degree) observed with equivalent concentrations of vectors [27]. Further work investigating the role of RPS3 in NF-κB activation led to the demonstration that *E. coli* O157:H7 NleH1 can inhibit RPS3 phosphorylation by IKK-β [45]. Inhibiting the phosphorylation of RPS3 restricts its translocation into the nucleus, reducing transcription of RPS3 dependant κB sites [27], [45], [46]. Regardless of this, transcription of genes controlled by non-RPS3 dependant κB sites can still occur, providing some additional reasoning as to why NleH1 and NleH2 did not significantly affect NF-κB activation in our assay. The kinase activity of NleH1 is required to inhibit RPS3 phosphorylation by IKK-β but it does not directly phosphorylate either protein. NleH also inhibits the pro-apoptotic pathway via its interaction with Bax Inhibitor 1 (BI-1) [28]. This interaction with BI-1 is dependent upon the C-terminal end of NleH but not its kinase activity; this region of NleH shares high protein identity with OspG, yet OspG does not interact with BI-1. It has yet to be elucidated whether NleH phosphorylates proteins other than itself. Overall, NleH is a multi-functional protein, a common trait for *E. coli* O157:H7 effector proteins.

This study highlights the need to not only determine the function of putative effector proteins but also how their expression is regulated in relation to the apparatus that secretes them. We provide further evidence that *nleH1* and *nleH2* are expressed under the same conditions that promote LEE expression but this expression is not strictly co-ordinated *in vitro*. Both *nleH* alleles are transcriptionally regulated by Ler and GrlA in EHEC in comparison to the post-translational regulation by LonP protease for *C. rodentium*, highlighting the importance of independently investigating the regulation of similar genes in related pathogens.

## Materials and Methods

### Bacterial strains and media

Strains used in this study are described in Table 1. Luria-Bertani broth and two defined media were used, Minimal Essential Media with HEPES modification (MEM; Sigma M7278), and Dulbelcco's Minimal Essential Media (DMEM; Sigma M5671). Glucose was added to the MEM to give a final concentration of 0.2%. Antibiotics were included where necessary at the following concentrations: 50 µg/ml kanamycin (Kan), 12.5 µg/ml chloramphenicol (Chl), 100 µg/ml ampicillin (Amp), 15 µg/ml gentamycin (Gent).

**Table 1:** Bacterial strains and plasmids used in this study.

Strain	Description	Reference/Source
TUV 93-0	EHEC O157:H7 strain EDL933; *stx* ^−^ derivative	[49]
ZAP198	EHEC O157:H7 strain NCTC 12900 *stx* ^−^	[50]
ZAP198Δ*ler*	Unmarked *ler* deletion mutant in strain ZAP198	[50]
ZAP198Δ*grlA*-Tn-Kan	*grlA* transposon mutant in EHEC strain ZAP198 (Tn5)	[51]
BW25113	*E. coli* K-12.	[52]
BW25113 *rpos*:Kan	BW25113 Keio mutant of *rpoS*.	[52]
MC1000	*araD139 Δ(araABC-leu)7679 galU galK Δ(lac)X74 rpsL thi*.	[53]
VS184	MC1000Δ*qseC*	[54]
XL1-Blue	*recA1 endA1 gyrA96 thi-1 hsdR17 supE44 relA1 lac* [F′ *proAB lacI* ^q^ZΔM15 Tn10 (Tet^r^)]	Stratagene
**Plasmid**		
pAJR70	pACYC eGFP	[30]
pAHE8	pAJR70 Ω [BamHI KpnI −531 bpNleH1_291::*gfp*]	This study
pAHE18	pAJR70 Ω [BamHI KpnI −120 bpNleH1_291::*gfp*]	This study
pAHE19	pAJR70 Ω [BamHI KpnI −283 bpNleH1_291::*gfp*]	This study
pAHE20	pAJR70 Ω [BamHI KpnI −113 bpNleH2_293::*gfp*]	This study
pAHE21	pAJR70 Ω [BamHI KpnI −291 bpNleH2_293::*gfp*]	This study
pAHE22	pAJR70 Ω [BamHI KpnI −655 bpNleH2_293::*gfp*]	This study
pAJR75	pAJR70 Ω [BamHI KpnI −442 bp including *LEE5* promoter cloned in frame 5′ to *egfp*	Roe 2004
pAJR145	pACYC rpsm::GFP+	Roe 2004
p*lacZ*	β-galactosidase enzyme constitutively produced by mammalian expression vector	Stratagene
NF-κB *luc*	Firefly luciferase gene under the control of canonical NF-κB promoter	Stratagene
pCMVTag3A	N-terminal myc tagging mammalian expression vector	Stratagene
pCMV-NleH1	pCMVTag3A Ω [PstI HindIII - NleH1 ORF]	This study
pCMV-NleH2	pCMVTag3A Ω [PstI HindIII – NleH2 ORF]	This study
pCMV-NleH1(D258)	Site directed mutant (SDM) of NleH1 residue D258 to alanine (A)	This study
pCMV-NleH1(E173)	SDM of NleH1 residue E173 to alanine (A)	This study
pCMV-NleH1(K159A)	SDM of NleH1 residue K159 to alanine (A)	This study
pJ201-OspG	OspG ORF with PstI+HindIII sites	DNA2.0/this study
pCMV-OspG	pCMVTag3A Ω [PstI HindIII - OspG ORF]	This study

### Plasmid-based translational fusion construction and NleH expression constructs

Three promoter lengths and the entire coding sequence for NleH1 and NleH2 were amplified from strain TUV-930 and cloned into pAJR70 to create pAHE8, pAHE18-22 (Table 1). Figure S1 shows the putative promoter regions that were amplified and cloned to create the constructs.

Expression plasmids for NleH1 and NleH2 were made as shown in Table 1. Site directed mutants were made using the Quick Change 1 mutagenesis system (Stratagene).

### Analysis of bacterial fluorescence

Constructs pAHE8 and 18–22 were assessed by growing ZAP193, and mutant where indicated, transformants overnight in LB Chl then the next morning diluted to a final OD_600_ of 0.08 into minimal media the next morning. Typically, 20 ml was cultured in Erlenmeyer flasks shaken at 180 rpm, 37°C. At regular intervals, 1 ml of culture was removed from the flask and 200 µl aliquots were analysed in triplicate with a fluorescent plate reader (Fluorostar Optima; BMG) at 37°C. For any strain and media combination, the promoterless gfp plasmid pAJR70 was included as a control. Fluorescence was plotted against OD_600_ using Graphpad Prism 5 software and a line of best fit obtained. Using this method, data were corrected for background fluorescence. At least three biological replicates were carried out for each experiment. To measure single-cell expression by fluorescence microscopy, strains were grown in MEM and at OD_600_ 0.8 a 100 µl aliquot was removed and diluted 1∶1 in 4% paraformaldehyde. 20 µl was dried onto a microscope slide and EspA filaments stained as described previously [30]. The slides were examined by fluorescence microscopy on a Zeiss Axioskop M1 fluorescence microscope, using the appropriate filter sets and a Z-stack of 16 images was captured at a spacing of 0.15 µm using Volocity software (PerkinElmer). These images were used to create a composite image that reduced the spatial effects of bacteria in different focal planes. The average gfp units per voxel (cubic pixel) was quantified, for at least 100 bacteria with a minimum volume of 4 µm^3^, using Volocity Quantification software (Perkin-Elmer). These values were exported and plotted in GraphPad Prism 5 (GraphPad Software, USA).

### Expression on contact with EBL cell lines

Embryonic bovine cell (German Collection of Microorganisms and Cell Cultures, no. ACC192, provided by Dr Arvind Mahajan, University of Edinburgh) were prepared and cultured as described previously [47]. The ZAP193 strain transformed with the appropriate GFP reporter plasmids was cultured in MEM-HEPES to OD_600_ 0.8 at 37°C, added to the multichamber slide, and centrifuged onto the EBL cells (1000×*g*) for 5 min (Time 0). The cells were stained at intervals by removal of the culture and addition of CellMask™ Deep Red plasma membrane stain (Invitrogen) before fixation with 4% paraformaldehyde. Time points analysed were 0, 5, 30, 180, and 420 min after addition. Immunostaining was performed using Mast α-O157 antibody and α-rabbit AlexaFluor-555 conjugate secondary antibody. Fluorescence analysis using Zeiss and Volocity software was then performed as described previously.

### RT-PCR analysis

Triplicate ZAP193, ZAP193ΔgrlA and ZAP193Δler were cultured in MEM-HEPES media to an OD_600_ = 1.2. Bacterial pellets were suspended in RNAProtect Bacteria Reagent (Qiagen). Total RNA was extracted using Qiagen RNeasy Mini kit and cDNA synthesis was carried out using a Qiagen QuantiTect™ Reverse Transcription kit. Duplicate qPCRs were carried out using a Qiagen Quantifast™ SYBR® green PCR kit and Stratagene MX3000 and primers listed in Table S2. All the experiments were performed according to manufacturer's instructions.

### Transfection of HEK293T cells

HEK293T cells [29] were cultured in DMEM (Invitrogen 21989) supplemented with 1 mM L-Glutamine, 10% foetal calf serum and penicillin/streptomycin. HEK293T cells were plated at a density of 5×10^4^ cells per well of a 24 well plate and, once 90% confluent, transfected with 0.4 µg of control (pCMV) or expression plasmid (pCMVNleH1, pCMVNleH1K159A, pCMVNleH1E173A, pCMVNleH1D258A pCMVNleH2 or pCMVOspG (DNA 2.0), 0.4 µg NF-κB luciferase reporter plasmid (Stratagene) and 0.1 µg β-galactosidase plasmid (a gift from Dr. Alison Michie). TNF-α (25 ng/ml) was added 40 hours after transfection. 24 hours after stimulation, cells were washed twice with PBS before lysates prepared and analysed with Dual-Light® System (Applied Biosystems). Luciferase activity was determined and normalized to β galactosidase activity as described [48]. Each assay was performed in triplicate, measured in duplicate and repeated three times.

## Supporting Information

Figure S1 **655 bp of upstream untranslated region (UTR) of** ***E. coli*** **O157:H7 EDL933 NleH1 (z0989) and NleH2 (z6021) were aligned using ClustalW.** Primers designed to construct translational fusions to GFP are labelled; green for NleH1 and blue for NleH2.
(TIFF)

Figure S2 **pAHE8 (NleH1-GFP) and pAHE22 (NleH2-GFP) were transformed into ZAP193 (A), ZAP193Δler (B) and ZAP193ΔgrlA (C) and at OD_600_ = 0.8 fixed with 4% paraformaldehyde onto a microscope slide.** Expression of NleH-GFP (green) and immunostained EspA filaments (AlexaFluor555; red) were observed using the appropriate filter sets. Micrographs are the composite image from 16 z-slices with 0.15 µm spacing.
(TIFF)

Table S1 **Sequences containing promoter regions were obtained from coliBASE and aligned using ClustalW.**
(DOC)

Table S2 **Oligonucleotides used in this study.**
(DOCX)
