# Supplementary figures and images for: Expression and Regulation of the Escherichia coli O157:H7 Effector Proteins NleH1 and NleH2
Source: PLoS One. 2012 Mar 12;7(3):e33408. doi: 10.1371/journal.pone.0033408 (PMC3299786; doi:10.1371/journal.pone.0033408)

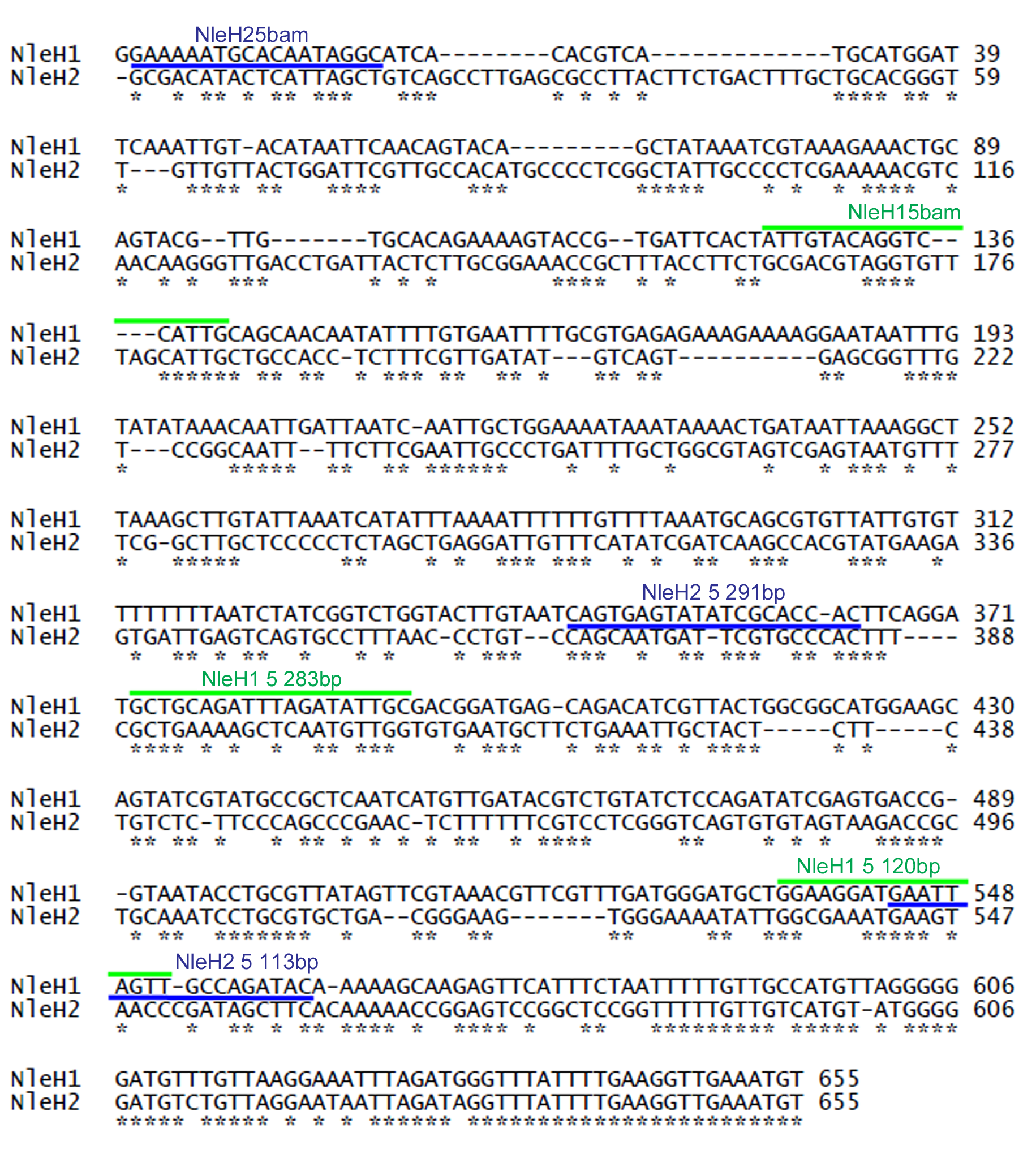

Supplement: Figure S1 — 655 bp of upstream untranslated region (UTR) of E. coli O157:H7 EDL933 NleH1 (z0989) and NleH2 (z6021) were aligned using ClustalW. Primers designed to construct translational fusions to GFP are labelled; green for NleH1 and blue for NleH2. (TIFF) [file pone.0033408.s001.tiff]

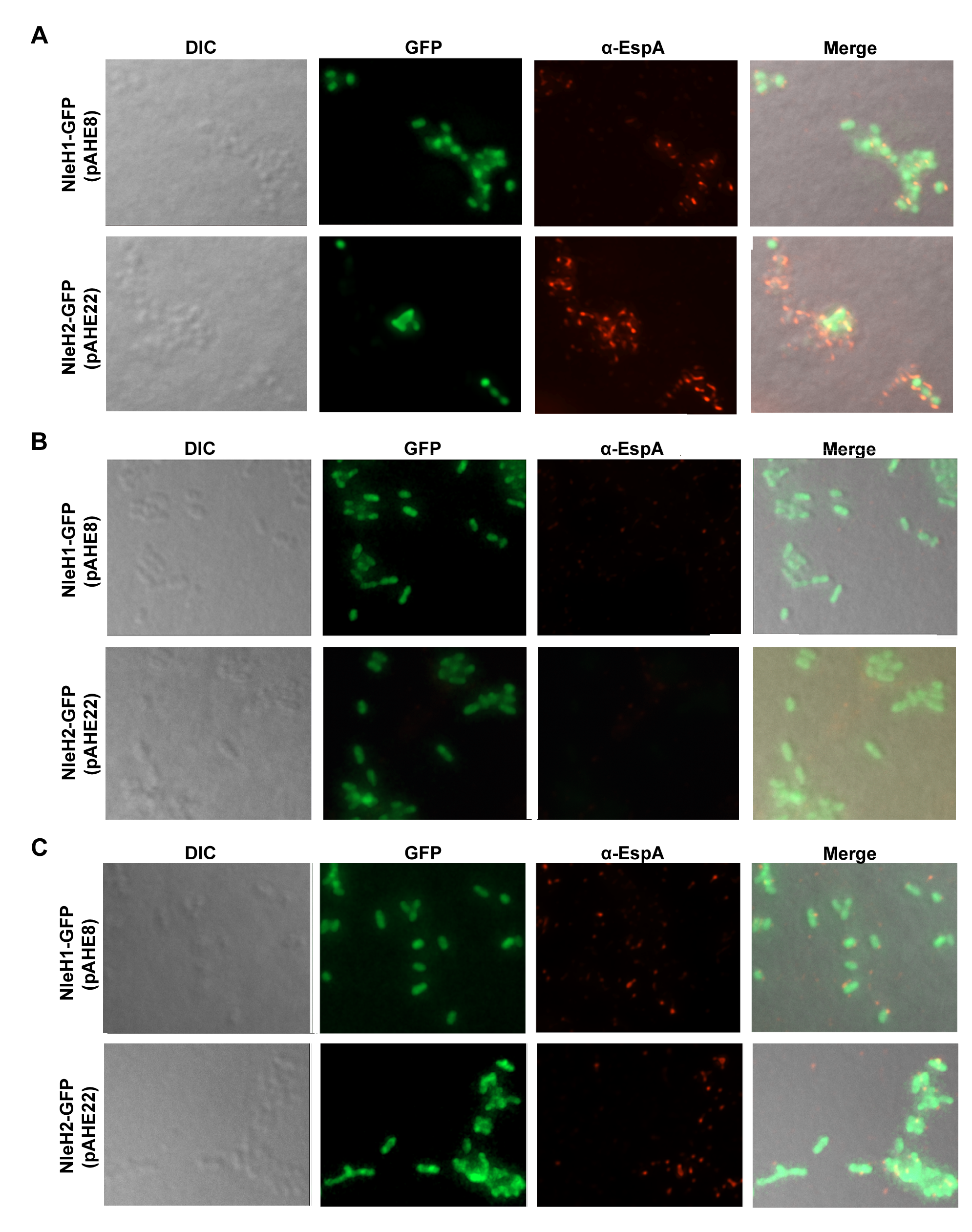

Supplement: Figure S2 — pAHE8 (NleH1-GFP) and pAHE22 (NleH2-GFP) were transformed into ZAP193 (A), ZAP193Δler (B) and ZAP193ΔgrlA (C) and at OD600 = 0.8 fixed with 4% paraformaldehyde onto a microscope slide. Expression of NleH-GFP (green) and immunostained EspA filaments (AlexaFluor555; red) were observed using the appropriate filter sets. Micrographs are the composite image from 16 z-slices with 0.15 µm spacing. (TIFF) [file pone.0033408.s002.tiff]
