# Supplementary material for: Expression and Regulation of the Escherichia coli O157:H7 Effector Proteins NleH1 and NleH2
Source: PLoS One. 2012 Mar 12;7(3):e33408. doi: 10.1371/journal.pone.0033408 (PMC3299786; doi:10.1371/journal.pone.0033408)
Supplement: Table S1 — Sequences containing promoter regions were obtained from coliBASE and aligned using ClustalW. (DOC) [file pone.0033408.s003.doc]

Table S1

| Upstream Sequence 1 | Upstream Sequence 2 | Alignment Score (ClustalW2) |
| --- | --- | --- |
| EPECNleH1 | EHECNleH1 | 51 |
| EPECNleH1 | CRODNleH | 50 |
| EPECNleH1 | EPECNleH2 | 53 |
| EPECNleH1 | EHECNleH2 | 53 |
| EHECNleH1 | CRODNleH | 53 |
| EHECNleH1 | EPECNleH2 | 53 |
| EHECNleH1 | EHECNleH2 | 53 |
| CRODNleH | EPECNleH2 | 52 |
| CRODNleH | EHECNleH2 | 53 |
| EPECNleH2 | EHECNleH2 | 99 |
