# Supplementary material for: Expression and Regulation of the Escherichia coli O157:H7 Effector Proteins NleH1 and NleH2
Source: PLoS One. 2012 Mar 12;7(3):e33408. doi: 10.1371/journal.pone.0033408 (PMC3299786; doi:10.1371/journal.pone.0033408)
Supplement: Table S2 — Oligonucleotides used in this study. (DOCX) [file pone.0033408.s004.docx]

Table S2

| Primer | Sequence |
| --- | --- |
| nleH15bam | CGGGATCCATTGTACAGGTCCATTGC |
| nleH1pro23kpn | CGGGTACCAGGCGAAGTCAGGTTTCTGGT |
| nleH1ser291kpn | CGGGTACCACTAATAAGATCTTGCTTTCC |
| nleH25bam | CGGGATCCGCGACATACTCATTAGCT |
| nleH2ser15kpn | CGGGTACCAGAATTCCATGAACATCCCAA |
| nleH2leu293kpn | CGGGTACCCAGCTTTCCTCCGTGATAAGA |
| nleH1 100bp | cggatccggaaggatgaattagttgcc |
| nleH1 250bp | cgggatccgctgcagatttagatattgc |
| nleH2 100bp | cgggatccgaagtaacccgatagcttc |
| nleH2 250bp | cgggatccagcaatgattcgtgcccac |
| H1 RT F | CCG AGT GTG GAC TAT AAC AGG TTG |
| H1 RT R | TCG TTG TCA CCT CTT CAT TGC |
| H2 RT F | GCC GAG GGT TAG CAA CAA TA |
| H2 RT R | ACG AAC TTC GCT TGT CAC CT |
| GapA RT F | GGGACGAAGTTGGTGTTGAC |
| GapA RT R | AACCACTTTCTTCGCACCAG |
| Tir RT F | CCATGGAGAGCAGACGTAGCT |
| Tir RT R | GCGAATCATGCAGCGA |
